# Supplementary material for: Overexpression of Lolium multiflorum LmMYB1 Enhances Drought Tolerance in Transgenic Arabidopsis
Source: Int J Mol Sci. 2023 Oct 18;24(20):15280. doi: 10.3390/ijms242015280 (PMC10607481; doi:10.3390/ijms242015280)
Supplement: Supplementary file 1 [file ijms-24-15280-s001.zip › Supplementary Figures S1-S4.pdf]

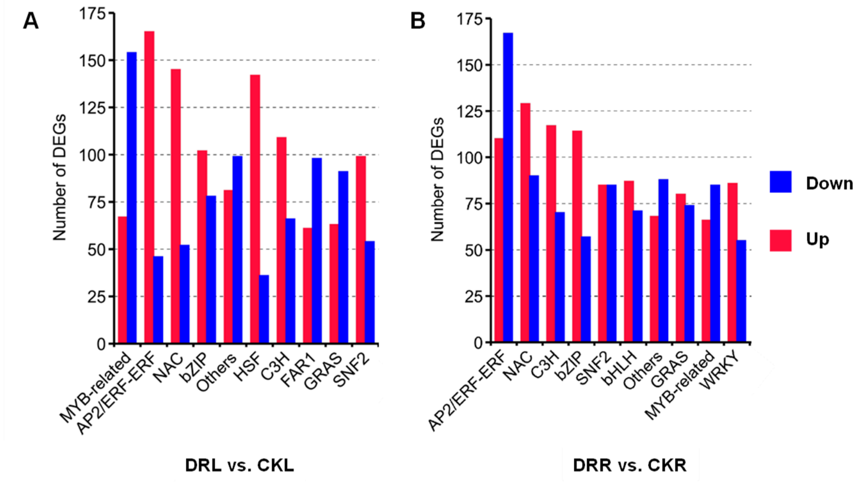

Figure S1: Heat maps of LmMYB1 and drought stress-related genes from transcriptome data. (**A**,**B**) stands for comparative combination DRL vs. CKL (**A**) and DRR vs. CKR (**B**), respectively. Blue represents genes that are down-regulated, while red represents genes that are up-regulated.

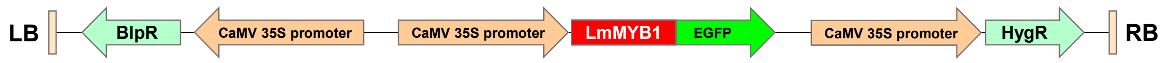

Figure S2: Vector construction of *LmMYB1*.

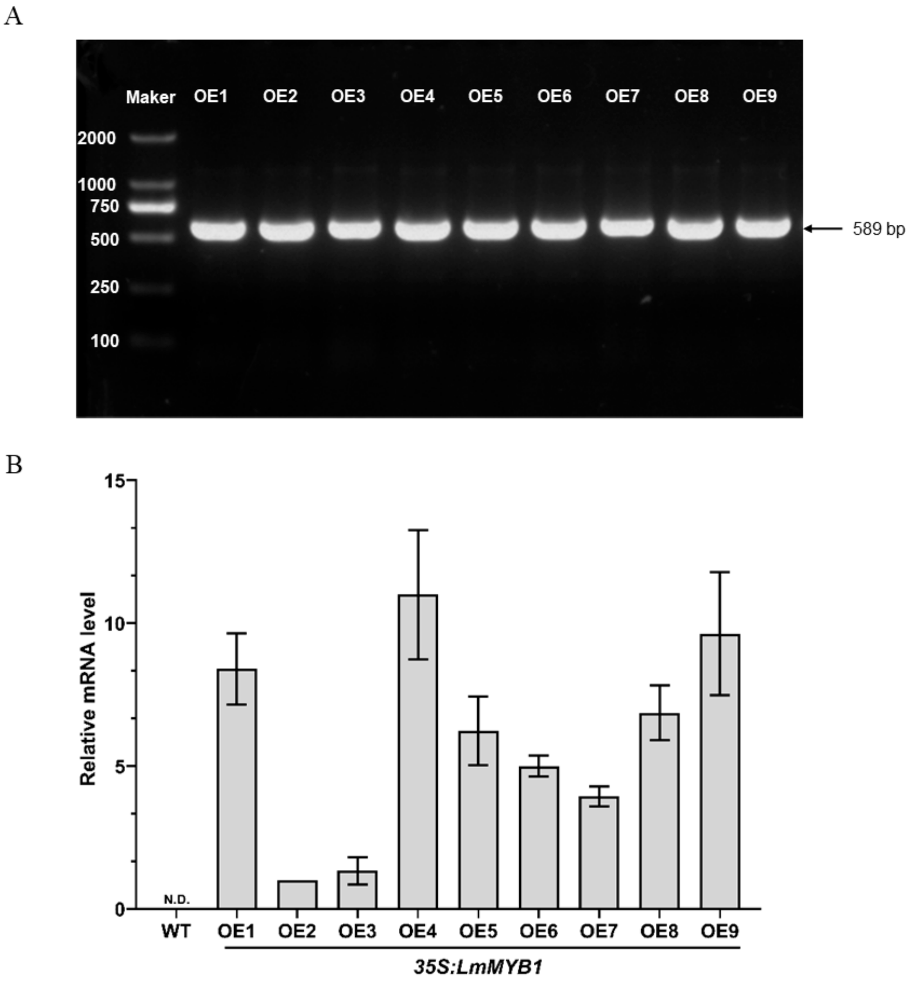

Figure S3: Validation of transgenic *Arabidopsis*. (**A**) DNA detection of T1 generation of transgenic *Arabidopsis* plant via RT-PCR. The target gene for positive plants screening is hygromycin phosphotransferase (HPT, 589 bp). (**B**) The expression of *LmMYB1* in transgenic *Arabidopsis* lines was validated through qRT-PCR.

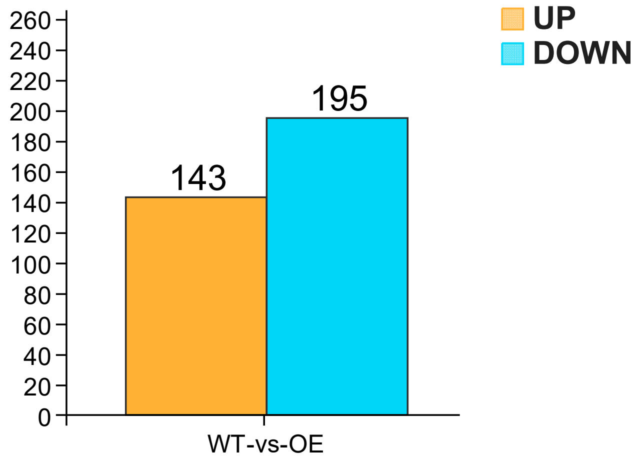

Figure S4: Histogram of the number of differentially expressed genes.
